# Cardiovascular magnetic resonance imaging derived septal curvature in neonates with bronchopulmonary dysplasia associated pulmonary hypertension

**DOI:** 10.1186/s12968-020-00643-x

**Published:** 2020-07-23

**Authors:** Paul J. Critser, Nara S. Higano, Sean M. Lang, Paul S. Kingma, Robert J. Fleck, Russel Hirsch, Michael D. Taylor, Jason C. Woods

**Affiliations:** 1 Heart Institute, Cincinnati Children’s Hospital Medical Center, Cincinnati, OH USA; 2 Center for Pulmonary Imaging Research, Cincinnati Children’s Hospital Medical Center, Cincinnati, OH USA; 3 Department of Pediatrics, University of Cincinnati College of Medicine, Cincinnati, OH 45229 USA; 4 Division of Neonatology and Pulmonary Biology, Cincinnati Children’s Hospital Medical Center, Cincinnati, OH USA; 5 Department of Radiology, Cincinnati Children’s Hospital Medical Center, 3333 Burnet Avenue, ML 5033, Cincinnati, OH 45229 USA

**Keywords:** Bronchopulmonary dysplasia, Cardiovascular magnetic resonance, Interventricular septal curvature, Neonatal lung disease, Pulmonary hypertension

## Abstract

**Background:**

Bronchopulmonary dysplasia (BPD) associated with pulmonary hypertension (PH) is a significant source of morbidity and mortality in premature infants. Recent advances have allowed the use of cardiovascular magnetic resonance (CMR) in the assessment of respiratory and cardiac disease in infants with BPD. In adults and older pediatric patients, decreased CMR interventricular septal curvature correlates with increased mean pulmonary artery pressure and pulmonary vascular resistance. The current study sought to determine the relationship of CMR derived septal curvature in neonates with BPD and BPD-PH with a need for PH therapy.

**Methods:**

Forty moderate or severe BPD and 12 mild BPD or control infants were imaged without contrast between 38 and 47 weeks post-menstrual age on a neonatal-sized, neonatal intensive care unit-sited 1.5 T CMR scanner. CMR indices including eccentricity index (CMR-EI) and septal curvature were measured and compared to BPD severity and clinical outcomes including hospital length of stay (LOS), duration of respiratory support, respiratory support level at discharge and PH therapy.

**Results:**

CMR-EI was directly associated and septal curvature was inversely associated with BPD severity. In a univariate analysis, CMR-EI and septal curvature were associated with increased hospital LOS, duration of respiratory support, respiratory support at hospital discharge, and need for PH therapy. In multivariable analysis CMR-EI was associated with hospital LOS and duration of respiratory support and septal curvature was associated with respiratory support at hospital discharge. Septal curvature was the only clinical or CMR variable associated with need for PH therapy (*R*^*2*^ = 0.66, *p* = 0.0014) in multivariable analysis demonstrating improved discrimination beyond CMR-EI.

**Conclusions:**

CMR derived septal curvature correlates significantly with clinical outcomes including hospital LOS, duration of respiratory support, respiratory support level at hospital discharge, and PH therapy in neonates with BPD and BPD-PH. Further, CMR derived septal curvature demonstrated improved discrimination of need for PH therapy and respiratory support at discharge compared to clinical variables and other CMR indices, supporting septal curvature as a non-invasive marker of PH in this population with potential to guide management strategies.

## Background

Bronchopulmonary dysplasia (BPD) is a significant contributor to morbidity and mortality in premature infants [1–3]. Alterations in lung development in BPD infants are associated with pulmonary vascular disease and pulmonary hypertension (PH). BPD associated PH (BPD-PH) is associated with increased BPD severity, short and late term respiratory outcomes and mortality, and PH secondary to BPD is associated with increased mortality compared to other etiologies of PH [2, 4–11].

While invasive hemodynamic assessment with cardiac catheterization remains the gold standard for diagnosis of PH, it may be challenging in critically ill neonates. Increasingly, cardiovascular magnetic resonance (CMR) is used for non-invasive assessment of PH in adult and older pediatric populations and recent advances in CMR pulse sequences and equipment have allowed for assessment of the lung parenchyma and cardiac morphology of infants with BPD and BPD-PH [12–20].

In particular CMR derived interventricular septal curvature, which provides a quantitative assessment of the interventricular septum throughout the cardiac cycle, has been associated with mean pulmonary artery pressure and pulmonary vascular resistance in adult and pediatric patients [17, 21–24]. However, no studies have assessed the relationship of septal curvature with PH in the BPD population.

The current study sought to assess the correlation of CMR derived septal curvature with short term clinical outcomes in neonates with BPD and BPD-PH. We hypothesized that decreased septal curvature would be associated with BPD severity, need for respiratory support and pulmonary vasodilator therapy, and demonstrate improved discrimination compared to other CMR indices.

## Methods

### Study subjects

Neonates included in the study were enrolled with Institutional Review Board approval. Inclusion criteria for neonates with BPD included BPD diagnosis per the 2001 National Institute of Child Health and Human Development and National Heart, Blood, and Lung Institute consensus definition [25] and post-menstrual age of 48 weeks or less at the time of CMR. Inclusion criteria for control infants included full-term birth (≥ 37 weeks gestational age) or pre-term birth in the absence of BPD, no clinically significant lung disease, and post-menstrual age 48 weeks or less at CMR. Control subjects were comprised of infants with primary neurologic or gastrointestinal diagnosis. Exclusion criteria for all study subjects consisted of evidence of significant genetic abnormalities or congenital malformations, evidence of respiratory infection at time of CMR, and standard CMR exclusion criteria.

### CMR protocol

Research CMR acquisitions were performed using a 1.5 T scanner (originally manufactured by ONI Medical Systems; currently GE Healthcare, Waukesha, Wisconsin, USA) sited in the neonatal intensive care unit [26]. CMR studies were conducted free breathing and without sedation unless indicated as part of their clinical care. CMR sequences used for cardiac analysis included a short-axis, retrospective electrocardiogram-gated balanced steady-state free-precession (bSSFP) imaging acquisition and an axial electrocardiogram-triggered double inversion-recovery fast spin echo acquisition. As previously described typical short-axis bSSFP acquisition parameters included: echo time = 1.6 ms; repetition time = 3.7 ms; flip angle = 45 degrees; effective temporal resolution = 20 ms; field of view 28–32 cm; pixel resolution 1.09–1.25 mm; slice thickness = 5–6 mm; number of averages = 3; and estimated scan time = 2 min and typical fast spin echo acquisition parameters included: echo time = 43.0 ms; repetition time = 736.2 ms; flip angle = 90 degrees; field of view 16–17 cm; pixel resolution 0.63–0.66 mm; slice thickness = 4 mm; number of averages = 3; and estimated scan time = 3 min [20].

### CMR image analysis

**Fig. 1:**
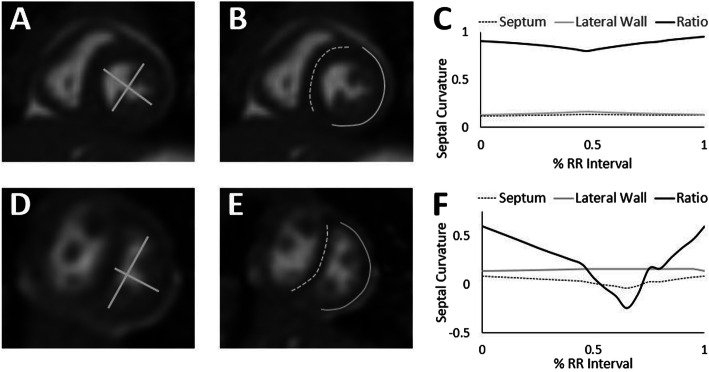
Cardiovascular magnetic resonance (CMR) eccentricity index (EI) and septal curvature for patients without septal flattening (a, **b**, **c**) and with septal flattening (**d**, **e**, **f**). CMR eccentricity index was derived as the ratio of the lateral to anterior-posterior diameter of the left ventricle (LV) at end systole (**a**, **d**). CMR septal curvature was derived as the ratio of curvature of the septum (dashed line) and lateral wall (solid line) throughout the cardiac cycle (**b**, **e**); CMR images (**b**, **e**) shown here demonstrate the time-point at which minimum septal curvature occurred in the two patients. Septal curvature across the cardiac cycle for the septum (dashed line), lateral wall (grey line) and ratio of septum to lateral wall (black line, **c**, **f**)

The right ventricle (RV) and left ventricle (LV) were contoured throughout the cardiac cycle in the short axis stack. RV end systolic volume indexed to body surface area (RVESVi), RV end diastolic volume indexed to body surface area (RVEDVi), RV mass indexed to body surface area, LV end systolic volume indexed to body surface area (LVESVi), LV end diastolic volume indexed to body surface area (LVEDVi), LV and RV ejection fraction (EF), and cardiac index (CI) were determined (cvi42, Circle Cardiovascular Imaging, Calgary, Canada). CMR-Eccentricity Index (CMR-EI) was measured as the ratio of the lateral to anterior-posterior diameter of the LV at end systole from the short axis stack at the level of the papillary muscles (Fig. 1).

Septal curvature was measured from the short axis stack at the level of the papillary muscles as previously described [17] using ImageJ (National Institutes of Health, Bethesda, Maryland, USA). A total of 8 points were placed along the LV lateral wall contoured from anterolateral to inferolateral septum and the interventricular septum contoured from RV insertion point to RV insertion point. Contours were propagated throughout the cardiac cycle and manually adjusted across 20 phases. Curvature analysis was performed for each phase of the cardiac cycle. The septal curvature was determined from the ratio of the curvature of the interventricular septum and LV lateral wall. Lower septal curvature values represent increased septal flattening with a negative septal curvature representing septal bowing into the LV. The minimum septal curvature was the minimum value of the septal curvature throughout the cardiac cycle (Fig. 1). All measurements were conducted by a single cardiologist. A second cardiologist analyzed 10 (25%) randomly selected studies, and interobserver and intraobserver intraclass correlation coefficients were determined.

### Statistical analysis

Univariate ANOVA tests were used to determine group differences in CMR indices, birth weight, gestational age, BPD severity, and short term clinical outcomes including length of stay (LOS), duration of respiratory support, respiratory support at hospital discharge, and need for PH therapy for three groups: subjects who did not receive PH therapy, patients who received PH therapy in the hospital, but not at discharge, and patients who received PH therapy in the hospital and at discharge. Univariate ANOVA tests were used to determine association of septal curvature with short term outcomes and of CMR indices and clinical variables with septal curvature. Mean and standard deviation are reported for parametric group data and mean and interquartile range for non-parametric group data.

Multivariable linear regression models were developed to assess the association of CMR indices and clinical variables with clinical outcomes using forward stepwise model with *p*-value ≤0.10 for inclusion in the original model and p-value ≤0.05 to remain in the final model. All analyses were performed in JMP 14.3 (SAS Institute Inc. Cary, North Carolina, USA).

## Results

### Patient demographics

Demographics for the cohort are shown in Table 1. The study cohort consisted of 52 neonates including 40 with moderate or severe BPD and 12 mild BPD or control infants. The median age at CMR was 41 weeks post-menstrual age [interquartile range (IQR) 39.6–42.5] and median heart rate was 157 beats per minute (IQR 143–166). CMR was completed in all infants without adverse events and septal curvature was measured in 46 (88%) infants. The interobserver intraclass correlation coefficient for septal curvature was 0.953 with 95% confidence interval 0.820–0.989 and the intraobserver intraclass correlation coefficient was 0.889 with 95% confidence interval 0.614–0.972.

**Table 1:** Demographic data for the study cohort

	No PH therapy	PH therapy in hospital	PH therapy at DC	*P* value
Birth weight (grams)	1236 ± 765	622 ± 162	651 ± 383	0.0143
Gestational age (weeks)	29.0 ± 4.9	25.9 ± 2.7	25.4 ± 2.0	0.0298
Severe BPD n (%)	13 (38)	8 (100)	10 (100)	0.0009
Hospital LOS (days)	144.8 ± 102.2	263.5 ± 141.5	297.1 ± 177.1	0.0021
Total Respiratory Support (days)	146.9 ± 148.9	350.3 ± 176.6	370.4 ± 228.5	< 0.001
Discharge Respiratory Support (Vent or death) n (%)	0 (0)	5 (62)	9 (90)	< 0.001

Data are mean ± standard deviation, unless otherwise noted

*Abbreviations*: *PH* pulmonary hypertension, *BPD* bronchopulmonary dysplasia, *LOS* length of stay

### Pulmonary hypertension therapy

Eighteen (35%) of the infants received PH therapy in the hospital. PH therapy was discontinued prior to discharge in eight (44%) infants treated with PH therapy in the hospital. Need for PH therapy was inversely correlated with birthweight and gestational age and directly correlated with hospital LOS, duration of total respiratory support, and discharge respiratory support.

CMR indices RV mass, CMR-EI, and septal curvature were associated with PH therapy. Higher RV mass (*p* = 0.04), CMR-EI (*p* < 0.001) and lower septal curvature (< 0.001) were associated with PH therapy on univariate analysis. CMR indices RVESVi, RVEDVi, LVESVi, LVEDVi, RVEF, LVEF, and CI were not significantly associated with need for PH therapy (Table 2). Clinical variables including gestational age (*p* = 0.03), birthweight (*p* = 0.01), and BPD severity (*p* = 0.004) were associated with need for PH therapy on univariate analysis. On multivariable analysis including clinical variables and CMR indices, only septal curvature was independently associated with PH therapy (Table 3). The median percent of the cardiac cycle for minimum septal curvature was 53% (IQR 42–67%), 54% (IQR 51–75%), and 60% (IQR 58–74%) in patients never treated with PH therapy, treated with PH therapy in hospital but not at discharge, and patients treated with PH therapy at discharge (*p* = 0.16).

**Table 2:** Cardiac MRI data for the study cohort

	N	No PH therapy	PH therapy in hospital	PH therapy at DC	*P* value
RVEDVi (ml/m2)	46	40.6 ± 10.7	37.7 ± 16.7	43.8 ± 8.5	0.56
RVESVi (ml/m2)	46	15.9 ± 5.0	15.0 ± 4.5	19.4 ± 6.5	0.17
RVEF (%)	46	60.6 ± 6.9	60.7 ± 6.2	56.6 ± 12.3	0.42
RV mass (g/m2)	46	13.1 ± 3.6	15.4 ± 5.7	16.7 ± 3.2	0.04
LVEDVi (ml/m2)	46	43.6 ± 9.7	43.1 ± 16.1	37.1 ± 7.7	0.26
LVESVi (ml/m2)	46	17.1 ± 6.0	16.4 ± 8.5	14.0 ± 3.1	0.41
LVEF (%)	46	61.4 ± 6.6	61.4 ± 5.7	62.9 ± 3.9	0.79
CI (L/min/m2)	46	4.3 ± 1.4	4.0 ± 1.4	3.7 ± 0.7	0.39
CMR-EI	48	1.09 ± 0.10	1.15 ± 0.15	1.32 ± 0.22	< 0.001
Septal Curvature	46	0.77 ± 0.18	0.60 ± 0.19	0.06 ± 0.3	< 0.001

Data are mean ± standard deviation

*Abbreviations*: *RVEDVi* RV end diastolic volume indexed to body surface area, *RVESVi* RV end systolic volume indexed to body surface area, *RVEF* RV ejection fraction, *LVEDVi* LV end diastolic volume indexed to body surface area, *LVESVi* LV end systolic volume indexed to body surface area, *LVEF* LV ejection fraction, *CI* cardiac index, *CMR* cardiovascular magnetic resonance, *EI* eccentricity index

**Table 3:** Predictors of short-term clinical outcomes in multivariable analysis

	*R*^*2*^	Parameter estimate ± SE	*P* value
**Hospital LOS**	0.49		
BPD Severity		−43.4 ± 17.4	0.0167
CMR-EI		343.87 ± 107.50	0.0026
**Length of Total Respiratory Support**	0.55		
Body weight		−0.072 ± 0.033	0.0324
BPD Severity		−57.1 ± 23.1	0.0177
CMR-EI		539.71 ± 142.8	0.005
**Discharge Respiratory Support**	0.47		
BPD Severity (severe vs moderate)		−2.1 ± 0.93	0.0245
Septal curvature		4.49 ± 1.49	0.0027
**PH Therapy**	0.66		
Septal curvature		11.37 ± 3.55	0.0014

*Abbreviations*: *LOS* length of stay, *BPD* bronchopulmonary dysplasia, *PH* pulmonary hypertension, *EI* eccentricity index

### Respiratory outcomes

The median hospital LOS was 166.0 days (IQR 109.5–237.5) and the median length of total respiratory support was 167 (86.0–313.5) days. On univariate analysis septal curvature was inversely associated with hospital LOS (*R*^*2*^ = 0.17, parameter estimate ± standard error = − 172.0 ± 56.6, and *p* value = 0.004), and length of total respiratory support (*R*^*2*^ = 0.29, parameter estimate ± standard error = − 305.3 ± 74.3, and p value = 0.0002). Including clinical variables and CMR indices in multivariable analysis hospital LOS was associated with BPD severity and CMR-EI, and length of total respiratory support was associated with BPD severity, body weight, and CMR-EI (Table 3). Twelve infants were discharged from the hospital on supplemental oxygen, 15 required tracheostomy, and two died of respiratory complications. Septal curvature was associated with level of discharge respiratory support on univariate analysis (*R*^*2*^ = 0.45, *p* value < 0.0001) and septal curvature and BPD severity were the only clinical or CMR variables significantly associated on multivariable analysis.

### Parameters associated with septal curvature

The following variables were associated with septal curvature on univariate analysis: birthweight (*R*^*2*^ = 0.17, *p* = 0.005), gestational age (*R*^*2*^ = 0.15, *p* = 0.007), BPD Severity (*R*^*2*^ = 0.17, *p* = 0.018), RV mass (*R*^*2*^ = 0.25, *p* = 0.0005), CMR-EI (*R*^*2*^ = 0.55, *p* = < 0.0001), RVEDVi (*R*^*2*^ = 0.10, *p* = 0.035), and RVESVi (*R*^*2*^ = 0.21, *p* = 0.002). On multivariable analysis including clinical variable and excluding CMR indices, gestational age was the only independent variable associated with septal curvature (*R*^*2*^ = 0.15, *p* = 0.007).

## Discussion

This study is the first to evaluate septal curvature in neonates with PH. CMR derived septal curvature was able to be determined in the vast majority of patients and was independently associated with level of respiratory support at hospital discharge and need for PH therapy, performing better than clinical and other CMR indices such as RV mass and CMR-EI. Initiation of PH therapy in this population is not currently standardized and often relies on qualitative assessment of clinical and echocardiographic data. These data suggest that septal curvature is a quantitative imaging marker that could allow for risk stratification and initiation of early therapy in neonates with BPD-PH.

Similar to previous studies, 35% of the cohort of BPD infants had PH with resolution of PH in 44% of neonates with BPD-PH [10, 11]. Additionally, BPD infants with PH had lower gestational age, lower birthweight, increased BPD severity, longer hospital LOS, duration of respiratory support, and increased level of respiratory support at hospital discharge compared to infants without PH consistent with prior studies in this population [4–6, 27]. BPD severity, gestational age, and birthweight were also associated with septal curvature in univariate analysis. Gestational age, which has previously been shown to correlate with BPD severity and CMR-EI, was the only clinical variable associated with septal curvature in multivariable analysis [20, 27].

Respiratory outcomes were correlated with CMR indices of septal flattening. In multivariable analysis, including clinical and CMR parameters, CMR-EI was associated with hospital LOS and duration of total respiratory support and septal curvature was associated with level of respiratory support at hospital discharge in addition to BPD severity. These data support an association of cardiac indices septal curvature and CMR-EI with short-term respiratory outcomes in BPD infants.

Septal curvature was the only independent variable associated with need for PH therapy in multivariable analysis. This is consistent with prior reports which have demonstrated that CMR derived minimum septal curvature and echocardiography derived septal curvature measured at end systole are associated with elevated RV pressure [17, 28, 29]. In the report by Pandya et al. CMR derived septal curvature correlated with mean pulmonary artery pressure and pulmonary vascular resistance in older pediatric PH patients [17]. In that cohort normal controls had septal curvature > 0.9, while PH patients had septal curvature < 0.8. Similarly in this study cohort all patients discharged on PH therapy had septal curvature < 0.5, while only two patients who never received PH therapy had septal curvature < 0.5. The septal curvature for the all infants in the present cohort was < 1.0, which is lower than CMR derived septal curvature reported in healthy children and may be due to younger age and BPD associated lung disease [17].

Previous reports have demonstrated that EI derived from echocardiography is higher in infants with persistent pulmonary hypertension and in infants with BPD-PH compared to controls [30, 31]. Additionally, CMR-EI has been shown to correlate with PH therapy in neonates with BPD-PH [20]. CMR-EI was modestly correlated with septal curvature in this cohort. Eccentricity index provides a quantitative assessment of interventricular septal flattening at the end of LV systole, while septal curvature provides a continuous assessment throughout the cardiac cycle. Patients with PH may have prolonged RV contraction resulting in maximal septal flattening (minimum septal curvature) after the end of LV systole [32]. Pandya et al. demonstrated that minimal septal curvature in pediatric PH patients occurred after aortic valve closure, during early LV diastole [17]. Indeed, in the current study the majority of patients receiving PH therapy had minimum septal curvature between 58 and 74% of the cardiac cycle. By capturing maximal septal flattening, minimum septal curvature may provide a more sensitive marker of PH.

While cardiac catheterization remains the gold standard for the diagnosis of PH, CMR offers an alternative non-invasive assessment of PH. Recent studies have correlated CMR metrics, including CMR derived septal curvature with invasive hemodynamic data in older pediatric and adult patients [17, 33]. This study suggests CMR derived septal curvature provides a non-invasive marker of PH in BPD infants which can be readily obtained from routine CMR protocols without the need for sedation associated with cardiac catheterization.

This study has several limitations including the single center study design and retrospective analysis, increased percentage of moderate to severe BPD infants, and the lack of normative CMR data in term and preterm infants without BPD. Additional studies are needed to define normative septal curvature in neonates and to confirm the associations in larger cohorts.

## Conclusions

In this study of neonates with BPD and BPD-PH, CMR derived septal curvature was associated with respiratory support at discharge and PH therapy with improved discrimination compared to other CMR indices including CMR-EI. These data support septal curvature as a non-invasive marker of PH in this population with potential to guide treatment strategies.

## Supplementary information

**Additional file 1 **: **Figure S1.** Representative cine images of infants without septal flattening (A) and with septal flattening (B).

## Data Availability

The datasets used and analyzed during the current study are available from the corresponding author on reasonable request.

## Abbreviations

BPD: Bronchopulmonary dysplasia
bSSFP: Balanced steady state free precession
CI: Cardiac index
CMR: Cardiovascular magnetic resonance
EF: Ejection fraction
EI: Eccentricity index
LOS: Length of stay
LV: Left ventricle/left ventricular
LVEDVI: Left ventricular end diastolic volume index
LVEF: Left ventricular ejection fraction
LVESVI: Left ventricular end systolic volume index
PH: Pulmonary hypertension
RV: Right ventricle/right ventricular
RVEDVI: Right ventricular end diastolic volume indexed to body surface area
RVEF: Right ventricular ejection fraction
RVESVI: Right ventricular end systolic volume index
